# Early photoreceptor assessment as a predictor for visual acuity gain after vitrectomy for macula-off retinal detachment

**DOI:** 10.1186/s40942-025-00722-0

**Published:** 2025-08-20

**Authors:** Lourdes Vidal-Oliver, Jorge Mataix-Boronat, Margot Mangen, Enrique Alfonso-Muñoz, Elena Palacios-Pozo, M. Jesus López-Prats, Carmen Desco

**Affiliations:** 1 Vitreoretinal Unit, Fundación Oftalmología Médica Comunidad Valenciana, Valencia, Spain; 2 Department of Medicine and Surgery, CEU Universities, Valencia, Spain

**Keywords:** Retinal detachment, Outer retinal integrity, External limiting membrane, Ellipsoid zone, Ellipsoid zone reflectivity

## Abstract

**Background:**

Visual recovery after macula-off rhegmatogenous retinal detachment (RRD) remains limited in many cases, largely due to photoreceptor damage. This study aimed to assess the predictive value of early outer retinal findings on optical coherence tomography (OCT) for visual acuity outcomes.

**Methods:**

A retrospective, longitudinal study was conducted on 106 eyes with macula-off RRD treated with vitrectomy and gas tamponade. The primary outcome was best-corrected visual acuity (BCVA) at six months postoperatively. Predictive variables included ellipsoid zone (EZ) and external limiting membrane (ELM) integrity, relative EZ reflectivity (rEZR) at one month, as well as age, time to surgery, macular status, baseline BCVA, and RRD extension. Associations were analyzed using linear regression models.

**Results:**

One-month ELM and EZ integrity were independent predictors of six-month BCVA after adjusting for rEZR and baseline LogMAR BCVA (ELM absence: ß=0.33; EZ absence: ß=0.20; both *p* < 0.04). rEZR increased significantly between one and six months (*p* < 0.05), stabilizing thereafter, and moderately correlated with BCVA (*r*=-0.6). Other variables were not significant in univariate analysis and were excluded from the multivariate model.

**Conclusions:**

Absence of the ELM at one month indicates poor visual prognosis, corresponding to an estimated four-line visual loss. rEZR may serve as a sensitive marker of photoreceptor metabolic recovery. These findings support the development of imaging-based deep learning models for visual outcome prediction in macula-off RRD.

## Background

Rhegmatogenous retinal detachment (RRD) is among the most prevalent retinal pathologies requiring prompt surgical intervention. The interval between symptom onset and surgery is a critical determinant of postoperative visual outcomes, with evidence indicating substantial photoreceptor degeneration occurring as early as three days after detachment [1]. Even with anatomically successful reattachment, visual recovery—particularly in cases involving foveal detachment—is frequently suboptimal and protracted [2, 3]. Patients often report persistent visual disturbances, including metamorphopsia and aniseikonia, which are believed to arise from microscopic displacements of photoreceptor cells [4]. These subtle misalignments disrupt the original cellular architecture, leading to functional impairment despite a seemingly normal postoperative fundus appearance [5]. 

Although high-resolution subcellular imaging is now feasible—such as that enabled by adaptive optics optical coherence tomography (AO-OCT)—, it is not yet widely available in clinical practice. Instead, conventional OCT systems provide valuable surrogate markers of photoreceptor integrity [6, 7]. Specifically, structures such as the external limiting membrane (ELM) and the ellipsoid zone (EZ) can be visualized and evaluated. Histologically, the ELM represents the processes of Müller cells and the tight junctions between photoreceptor inner segments, while the EZ corresponds to the mitochondria-rich portion of the photoreceptor inner segments [8, 9]. The structural integrity of these layers, as seen on OCT, serves as an indirect indicator of photoreceptor health. Furthermore, the reflectivity of the EZ (EZR) may correlate with the metabolic activity of photoreceptors [10]. Therefore, longitudinal assessment of EZR could provide more accurate insights into photoreceptor functional recovery following surgical repair.

Recently, Martins Melo and colleagues proposed a subclassification system for macula-off RRD, demonstrating that higher stages correlate with poorer visual outcomes. Notably, the presence of high frequency, high amplitude corrugations was identified as a critical threshold beyond which visual recovery is significantly compromised [11]. This classification has clinical utility in guiding preoperative counseling and managing patient expectations regarding visual prognosis. In the present study, we hypothesize that early assessment of the outer retina—specifically at one month postoperatively—may provide additional valuable prognostic information for predicting best-corrected visual acuity at mid-term follow-up, specifically at six months after surgery.

## Methods

### Study design and participants

This was a single-center, retrospective, longitudinal study including patients who underwent pars plana vitrectomy (PPV) with gas tamponade for RRD between 2022 and 2024 at a specialized referral center for retinal surgery.

Exclusion criteria included prior retinal surgery, presence of pathologic myopia beyond the stage of diffuse chorioretinal atrophy, any signs of epiretinal membrane at any visit, macular edema at any visit, postoperative outer retinal folds affecting the macula, or retinal re-detachment during follow-up, intraoperative internal limiting membrane (ILM) peeling, and persistent subfoveal subretinal fluid at the first postoperative visit. These conditions were excluded because they can independently affect visual acuity or alter outer retinal morphology on OCT, potentially confounding the predictive value of the biomarkers under study.

### Variables collected

Data were extracted from electronic medical records. Baseline variables included patient age, gender, lens status, symptom duration (patient-reported in days, primarily reflecting symptoms related to macular involvement), presence of pathologic myopia, preoperative best-corrected visual acuity (BCVA) in both eyes, macular status based on OCT, and extent and location of the RRD. Macular status was classified as < 3b or ≥ 3b based on previously published criteria [11, 12]. A retinal specialist assessed RRD extension (in clock hours) and location (quadrants involved) using ultra-widefield fundus imaging (Optomap, Optos, CA).

Intraoperative data included surgical technique (PPV alone or combined with scleral buckle) and type of intraocular gas used. Follow-up assessments were conducted at 1, 3, 6, and 12 months postoperatively and included BCVA, relative ellipsoid zone reflectivity (rEZR), and qualitative evaluation of EZ and ELM integrity from macular OCT scans.

### Outcomes

The primary outcome was BCVA at 6 months postoperatively. Key exploratory variables included: (1) ELM integrity, (2) EZ integrity and (3) relative EZ reflectivity at 1 month; (4) patient’s age, (5) time to surgery, (6) preoperative macular status, (7) preoperative BCVA and (8) extent of RRD.

Descriptive data on demographic and surgical characteristics, as well as longitudinal changes in BCVA and rEZR over the 12-month period, were also reported.

### Retinal imaging and biomaker assessment

All patients underwent ultra-widefield fundus photography and spectral-domain OCT imaging at each visit, including preoperative evaluation. OCT scans (6-mm radial, fovea-centered, averaging 32 frames) were acquired using the DRI-OCT Triton device (Topcon, Japan). We included only OCT scans with a signal strength > 40. Preoperative macular status was classified based on the grading system by Martins Melo et al. [12]

**Fig. 1:**
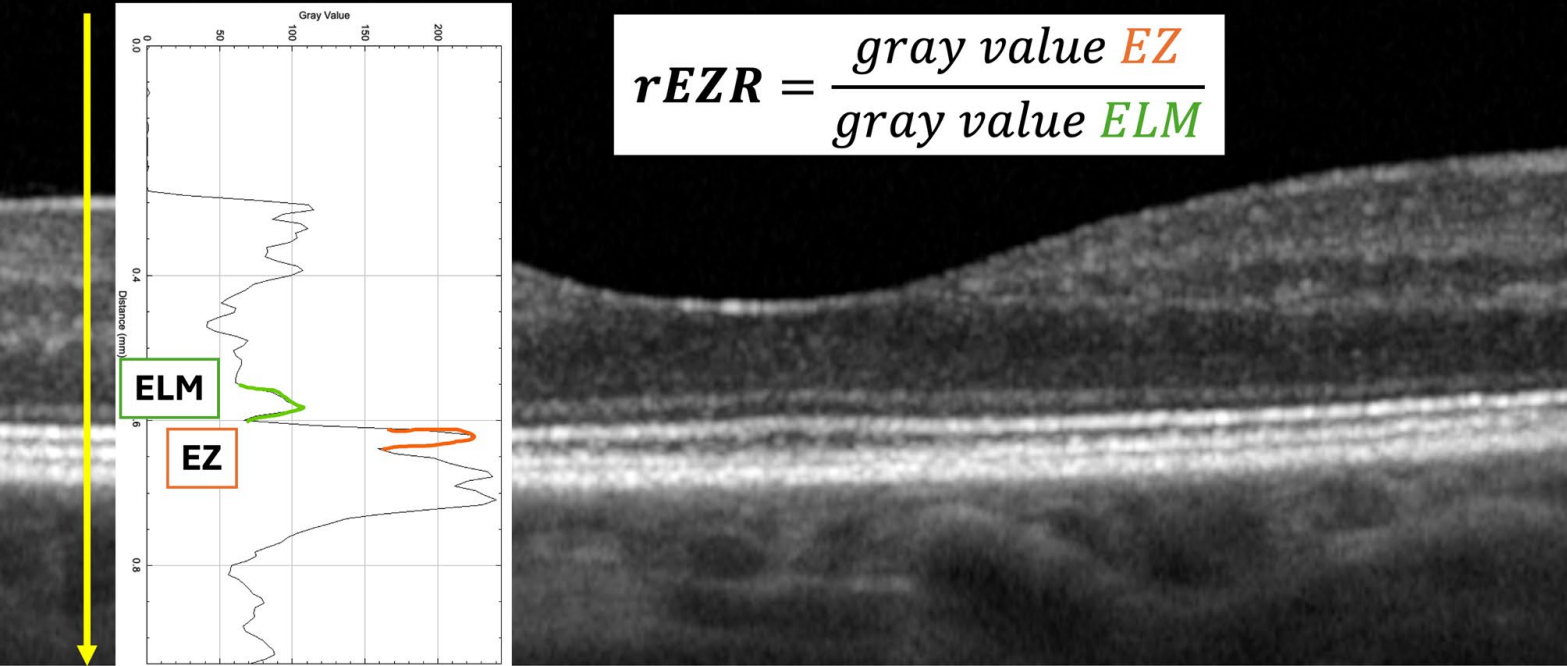
The relative ellipsoid reflectivity (rEZR) was calculated using the Plot Profile function of ImageJ (National Institutes of Health, Bethesda, MD, USA) as the ratio of peak intensities between the external limiting membrane (ELM) and the ellipsoid zone (EZ) reflectivity. This calculation was performed following the description of previous authors [25, 28]

The rEZR was calculated using ImageJ (National Institutes of Health, Bethesda, MD, USA). Grayscale intensity values were measured at the ELM and EZ using the Plot Profile function, and rEZR was defined as the ratio of EZ to ELM reflectivity (Fig. 1).

ELM and EZ integrity were independently graded at the 1-mm central fovea by two masked observers (LVO and CD) using three categories: continuous, discontinuous, or absent. Discrepancies were resolved by a third grader (JMB).

### Statistical analysis

Quantitative data were summarized as means ± standard deviations, while categorical variables were reported as percentages. Longitudinal changes in BCVA and rEZR were analyzed using mixed-effects models with correction for multiple comparisons. Chi-square tests, also adjusted for multiple comparisons, were used to compare qualitative variables (ELM and EZ integrity).

Associations between BCVA at 6 months and the exploratory variables were examined using linear regression models. Only variables that showed statistically significant associations in univariate analysis were included in the multivariate model. A *p*-value < 0.05 was considered statistically significant.

## Results

A total of 219 patients met the inclusion criteria. After excluding 113 eyes due to poor image quality (*n* = 21), significant postoperative cataract (*n* = 32), pathologic myopia at a stage beyond diffuse chorioretinal atrophy (*n* = 6), intraoperative ILM peeling or postoperative macular edema (*n* = 45), and persistent subfoveal fluid (*n* = 9), 106 eyes from 106 patients were included in the final analysis. The mean patient age was 63 years, and 71.7% were male. Detailed baseline characteristics are presented in Table 1.

**Table 1:** Patient characteristics

Age (mean, range)	62.9 (27–88)
Sex (M/F, %)	76/30 (72/28%)
Pathologic myopia (diffuse chorioretinal atrophy) (N, %)	36, 34%
Pseudophakic (N, %)	53, 50%
Baseline BCVA (logMAR)[mean, SD)	1.25 (0.87)
Macular status	
1	10 (9.4%)
2	22 (20.8%)
3a	29 (27.4%)
3b	22 (20.8%)
4	12 (11.3%)
5	1 (0.9%)
N/A	10 (9.4%)
Symptoms duration (days) [mean, SD)	10 (9.29)
Type of gas	
SF_6_	58 (54.7%)
C_3_F_8_	48 (45.3%)
Surgical technique	
PPV only	83 (81.1%)
PPV + buckle	28 (21.7%)

BCVA: best-corrected visual acuity, LogMAR: logarithm of the minimum angle of resolution, PPV: pars plana vitrectomy

Macular status using the classification proposed by Martins Melo et al. [12]

### Prediction of visual acuity at 6 months

In univariate analysis, all outer retinal parameters—rEZR, EZ integrity, and ELM integrity—along with baseline BCVA, were significantly associated with BCVA at six months. However, in the multivariate regression model, only EZ and ELM integrity at one month remained statistically significant predictors (Table 2).

**Table 2:** Linear regression analysis with best-corrected visual acuity six months after surgery as dependent variable

Univariate			Multivariate		
Dependent variable	Slope	95% CI	Dependent variable	Coefficient	95% CI
rEZR month 1	-0.26	-0.5 to -0.01*	rEZR month 1	-0.14	-0.38 to 0.11
EZ integrity month 1	0.08	0.02 to 0.17*	EZ integrity month 1 (absence)	0.20	0.07 to 0.33*
			EZ integrity month 1 (discontinuous)	0.09	-0.12 to 0.30
ELM integrity month 1	0.11	0.001 to 0.22*	ELM integrity month 1 (absence)	0.33	0.08 to 0.58*
			ELM integrity month 1 (discontinuous)	-0.19	-0.41 to 0.02
Baseline macular status	0.02	-0.16 to 0.21			
Baseline BCVA (logMAR)	0.08	0.01 to 0.16*		0.01	-0.06 to 0.08
Age	-0.002	-0.01 to 0.005			
Time to surgery (days)	0.004	-0.01 to 0.013			
RD extension (clock hours)	0.007	-0.02 to 0.04			

The multivariate model includes the independent variables that are statistically significant in univariate analysis. For both ELM and EZ integrity, the regression coefficients are expressed relative to the “continuous” category. *statistically significant, *p*-value < 0.05

rEZR: relative ellipsoid zone reflectivity, EZ: ellipsoid zone, ELM: external limiting membrane, BCVA: best-corrected visual acuity, LogMAR: logarithm of the minimum angle of resolution

ELM integrity was the strongest predictor (ß = 0.33; 95% CI: 0.08–0.58). Eyes with absent ELM one month after surgery had, on average, approximately three lines worse visual acuity at six months compared to those with continuous ELM, after adjusting for other factors. Additionally, the absence of the EZ was associated with a 0.20 LogMAR improvement (approximately two lines of worse vision; ß = 0.20; 95% CI: 0.07 to 0.33), compared with those eyes presenting with continuous EZ.

ELM disruption was less frequent overall, and all eyes with a preserved EZ also had intact ELM (data not shown). Frequencies are presented in Table 3. Other clinical variables—including baseline macular status, age, time to surgery, and detachment extent—were not significant in univariate analysis and were excluded from the multivariate model.

**Table 3:** Longitudinal changes in all the variables included in the multivariate analysis

	1 m	3 m	6 m	12 m	*p*-value 1 (1 m vs. 3 m)	*p*-value 2 (3 m vs. 6 m)	*p*-value 3 (6 m vs. 12 m)
BCVA (logMAR) [mean, sd]	0.40 (0.42)	0.25 (0.21)	0.17 (0.16)	0.27 (0.21)	0.006*	0.914	0.504
rEZR (AU) [mean, sd]	1.24 (0.31)	1.35 (0.23)	1.47 (0.21)	1.65 (0.20)	0.002*	0.049*	0.303
ELM integrity (%)					0.194	0.006*	0.140
Continuous	66%	76%	88%	91%			
Discontinuous	20%	17%	9%	8%			
Absent	14%	7%	0%	4%			
EZ integrity (%)					0.001*	0.020*	0.719
Continuous	34%	59%	76%	71%			
Discontinuous	42%	31%	21%	25%			
Absent	24%	10%	3%	4%			

Continuous variables were compared using myxed-effect analysis. Categorical variables were compared using chi-square test. All *p*-values shown have been adjusted for multiple comparisons

*statistically significant, *p*-values < 0.05

### Longitudinal analysis within the first year

Longitudinal assessment revealed that the most substantial changes in outer retinal biomarkers (rEZR, EZ, and ELM integrity) occurred within the first six months postoperatively, with no significant changes thereafter (Table 3).

**Fig. 2:**
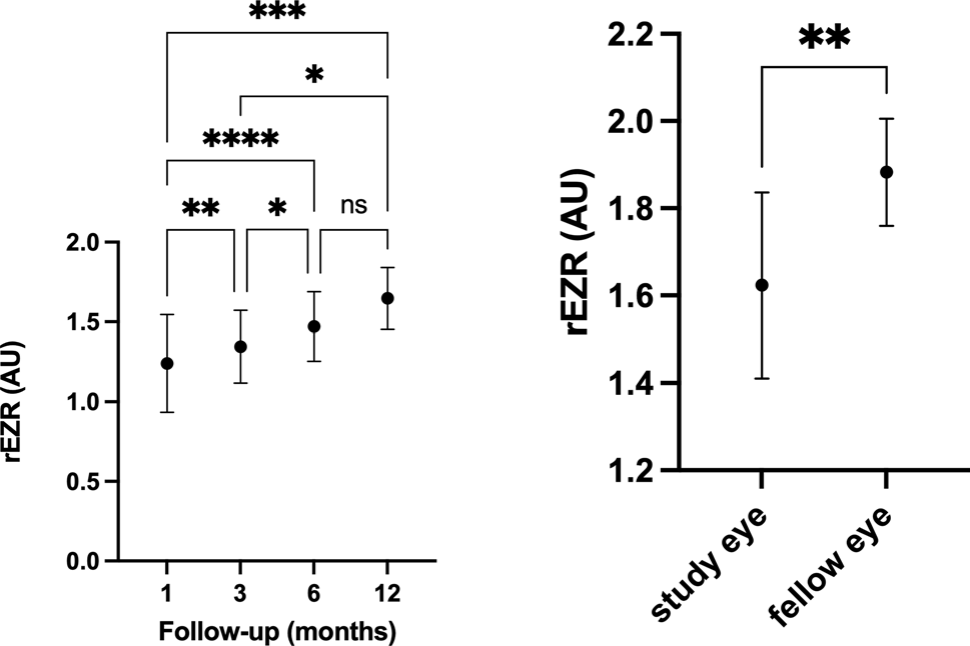
Changes in relative ellipsoid zone reflectivity (rEZR) over time (left) and a comparison of the final rEZR of the study eye and the fellow eye (right). rEZR increases with follow-up time, especially in the first 6 months. After that, the changes are not statistically significant. AU: arbitrary units. * *p*-value < 0.05

Importantly, even after structural restoration of the ELM and EZ, rEZR continued to provide meaningful information as a biomarker of photoreceptor recovery. rEZR values showed a moderate correlation with BCVA across the cohort (*r* = -0.61, *p* < 0.0001). Nonetheless, final rEZR measurements at one year did not reach the levels observed in the healthy fellow eyes (Fig. 2).

## Discussion

In this retrospective study, we demonstrated that evaluation of the outer retina using OCT, as early as one month after surgery, can effectively predict long-term visual acuity in patients with macula-off RRD.

Photoreceptor assessment has gained growing importance in both clinical and research settings. Notably, the loss of the EZ has recently been recognized by the FDA as a primary endpoint in clinical trials for age-related macular degeneration [13]. Given that RRD primarily affects the outer retina, numerous studies have investigated photoreceptor structure and function. However, significant heterogeneity exists in methodology—ranging from the definition of outer retinal bands to segmentation boundaries and quantification techniques [7, 14–18]. Parameters such as integrity, reflectivity, thickness, and area of loss have been measured using both manual and automated methods. This inconsistency highlights the need for standardized criteria and consensus definitions to enable the adoption of photoreceptor metrics as reliable surrogate endpoints in future clinical trials.

Among the evaluated biomarkers, ELM integrity emerged as the most significant early predictor of visual recovery. Clinically, ELM status can be assessed easily at one month postoperatively, offering a practical tool to guide visual prognosis. Our findings are in line with previous reports by Malosse et al. [16], who observed that ELM integrity predicts subsequent EZ restoration. Nevertheless, even after apparent anatomical recovery of the outer retinal bands on OCT, some patients continue to experience visual disturbances [2, 19]. The EZ corresponds to the photoreceptor inner segments, which are rich in mitochondria. Despite successful reattachment, photoreceptors may still suffer from structural misalignment or mitochondrial damage, which can reduce the reflectivity of the EZ band.

To investigate this further, we analyzed the rEZR, a potential biomarker of photoreceptor metabolic function [10]. Our longitudinal data showed that rEZR continues to change beyond the point of structural restoration, and moderately correlates with visual acuity. These findings suggest that rEZR could help identify patients who may experience ongoing functional recovery after the reappearance of the ELM and EZ bands. Importantly, rEZR values at one year remained lower than those in the unaffected fellow eye, indicating persistent subclinical dysfunction.

rEZR has been primarily studied in the context of age-related macular degeneration (AMD), using various methods such as normalized en face reflectivity maps [20, 21] and automated quantification from raw OCT data [10, 22] These studies consistently report lower rEZR values in diseased eyes, often associated with additional biomarkers such as reticular pseudodrusen or choriocapillaris flow deficits, supporting the use of EZR as a surrogate marker of photoreceptor integrity and metabolic impairment. While large-scale longitudinal datasets are not yet available, recent data from the MACUSTAR study showed a progressive decline in rEZR with advancing AMD stages [22]. In the context of retinal detachment, Quarta et al. reported significantly reduced normalized EZR in eyes with macula-on RRD compared to fellow eyes, suggesting subclinical pan-retinal metabolic and microvascular alterations even in the absence of overt macular involvement [21]. These findings align with our results, highlighting early photoreceptor dysfunction detectable by rEZR prior to visible structural changes. However, longitudinal data in RRD remain scarce.

Anatomically, the ELM consists of tight junctions between Müller cells and the inner segments of photoreceptors [23]. Disruption of the ELM may represent a structural threshold beyond which photoreceptor recovery becomes unlikely, reinforcing its prognostic significance.

While other studies have identified variables such as age, time to surgery, lens status, detachment extent, number of retinal breaks, and baseline macular status as predictors of visual outcomes [11, 24], we did not observe such associations in our cohort. This discrepancy may reflect differences in study populations and the inherent limitations of retrospective data. For instance, symptom duration was self-reported and clinical records often failed to distinguish between early symptoms (e.g., floaters) and the onset of macular involvement. Future prospective studies should define and capture the onset of macular detachment more precisely.

Limitations of our study include its retrospective design, a relatively small sample size, and the use of manual methods to assess outer retinal bands. Additionally, in some cases, residual intraocular gas—particularly in eyes treated with C_3_F_8_—was still present at the 1-month visit, which may have affected OCT signal quality and the assessment of ELM and EZ reflectivity. Although poor-quality images were excluded, this potential confounder should be considered when interpreting early postoperative measurements. Furthermore, axial length measurements were not available for all participants and were therefore not included in the analysis. Although we excluded highly myopic eyes with advanced chorioretinal atrophy, we acknowledge that subtle variations in axial length could influence image scaling. However, our use of point-based, longitudinal reflectivity measurements—focused on small foveal regions—has been shown to minimize the influence of axial length, which supports the robustness of our findings in this context [25]. 

Advanced imaging software now allows automated segmentation and quantification of outer retinal bands [26, 27]. Furthermore, future studies could improve rEZR quantification by analyzing raw OCT data prior to logarithmic transformation using existent advanced methods [28]. In the present study, rEZR was quantified in OCT images after logarithmic transformation, which are optimized for clinical visualization rather than quantitative analysis; this may affect intensity differences and limit accuracy [28]. 

Larger prospective studies incorporating automated tools, linear-scale reflectivity quantification and en face topographic mapping will be crucial for validating and expanding upon our findings.

## Conclusions

Our findings indicate that OCT-derived outer retinal biomarkers—including EZ, ELM and rEZR—at one month postoperatively provide valuable insights into visual prognosis following macula-off RRD. Among these, ELM absence was the most robust predictor of poor outcomes, while rEZR offers a sensitive metric to detect ongoing photoreceptor recovery after anatomical restoration.

## Data Availability

The datasets used and/or analysed during the current study are available from the corresponding author on reasonable request.

## Abbreviations

RRD: Rhegmatogenous retinal detachment
AO-OCT: Adaptive-optics optical coherence tomography
OCT: Optical coherence tomography
ELM: External limiting membrane
EZ: Ellipsoid zone
EZR: Ellipsoid zone reflectivity
PPV: Pars plana vitrectomy
ILM: Internal limiting membrane
BCVA: Best-corrected visual acuity
rEZR: Relative ellipsoid zone reflectivity
LogMAR: Logarithm of the mínimum angle of resolution
